# Patterns of the Predicted Mutation Burden in 19,778 Domesticated Barley Accessions Conserved Ex Situ

**DOI:** 10.3390/ijms25115930

**Published:** 2024-05-29

**Authors:** Yong-Bi Fu

**Affiliations:** Plant Gene Resources of Canada, Saskatoon Research and Development Centre, Agriculture and Agri-Food Canada, 107 Science Place, Saskatoon, SK S7N 0X2, Canada; yong-bi.fu@agr.gc.ca

**Keywords:** genotyping-by-sequencing, mutation, mutation burden, barley germplasm, germplasm management, germplasm conservation

## Abstract

Long-term conservation of more than 7 million plant germplasm accessions in 1750 genebanks worldwide is a challenging mission. The extent of deleterious mutations present in conserved germplasm and the genetic risk associated with accumulative mutations are largely unknown. This study took advantage of published barley genomic data to predict sample-wise mutation burdens for 19,778 domesticated barley (*Hordeum vulgare* L.) accessions conserved ex situ. It was found that the conserved germplasm harbored 407 deleterious mutations and 337 (or 82%) identified deleterious alleles were present in 20 (or 0.1%) or fewer barley accessions. Analysis of the predicted mutation burdens revealed significant differences in mutation burden for several groups of barley germplasm (landrace > cultivar (or higher burden estimate in landrace than in cultivar); winter barley > spring barley; six-rowed barley > two-rowed barley; and 1000-accession core collection > non-core germplasm). Significant differences in burden estimate were also found among seven major geographical regions. The sample-wise predicted mutation burdens were positively correlated with the estimates of sample average pairwise genetic difference. These findings are significant for barley germplasm management and utilization and for a better understanding of the genetic risk in conserved plant germplasm.

## 1. Introduction

The long-term conservation of more than 7 million plant germplasm accessions in 1750 genebanks worldwide [1] is a challenging mission [2]. Genetic erosion and vulnerability can occur within genebanks through mutation, genetic drift, and non-random viability selection [3,4,5,6]. Conserved germplasm will accumulate existing and new mutations created under long-term conservation conditions. However, little is known about the extent of deleterious mutations in conserved germplasm and the genetic consequences of the accumulated mutations on long-term germplasm conservation [3,7,8]. Theoretical studies predicted that mutation accumulation in regenerated seed collections could lower the viability of conserved germplasm [9,10]. Thus, the genetic cost for long-term germplasm conservation remains poorly understood [8].

Technical advances over the last 20 years in genome sequencing (e.g., wheat [11]), genetic load estimation in the human genome (e.g., [12,13]), and bioinformatics tools for predicting deleterious amino acid polymorphism (e.g., [14]), have made the genomic characterization of mutations in plant species with large and complex genomes technically and practically feasible [8,15,16]. Technically, the identification of deleterious variants across a sequenced genome was largely based on the prediction of deleteriousness at a nonsynonymous site change alone and/or in combination with the intensity of purifying selection inferred from phylogenetic restraints on the site. Many studies have been made to identify and characterize deleterious variants across plant genomes [15,17,18,19,20,21,22,23]. These studies have successfully demonstrated the usefulness of scanning and characterizing deleterious variants across plant genomes [15,24] and provided an informative estimation of mutation burdens (or precisely the extent of deleterious genetic variants across a genome) in conserved germplasm [8]. For example, we applied RNA-Seq technology [25] to characterize the extent and variation of deleterious and adaptive mutations in 490 individual plants representing barley, wheat, oat, soybean, maize, rapa, and sunflower collections in a seed genebank [8]. These collections were found to display significant variations in both the numbers of predicted deleterious mutations and the estimates of mutation burden. Similarly, variability in deleterious mutations was also observed among nine accessions of a barley collection conserved ex situ [26].

In this study, we took advantage of the barley genome and related genomic resources to predict sample-wise mutation burdens for 19,778 domesticated barley (*Hordeum vulgare* L.) accessions conserved ex situ. Milner and her colleagues [16] published the SNP genotype data from the application of genotyping-by-sequencing (GBS) technology to characterize the genetic diversity of 19,778 domesticated barley samples conserved in the Leibniz Institute of Plant Genetics and Crop Plant Research (IPK), Germany, and two other genebanks. We analyzed this large SNP genotype data set in combination with the related barley genomic resources created in our previous study [8] for three objectives to (1) identify deleterious SNPs, (2) estimate sample-wise mutation burdens, and (3) characterize the patterns of the predicted mutation burdens in the assayed barley samples. The revealed patterns of predicted mutation burdens are significant for barley germplasm management and utilization and for a better understanding of the genetic risk in conserved plant germplasm.

## 2. Results

### 2.1. Annotating SNPs and Identifying Deleterious SNP

Annotating the published 76,102 SNPs [16] across the barley genome using the stand-alone Ensembl Variant Effect Predictor (VEP) revealed that 74,856 SNPs were associated with 16 consequence classes; 12,783 SNPs were missense_variant; and 1626 SNPs were the variants associated with loss of function based on the total count of variants from seven annotation classes (854 splice_region_variants; 356 stop_gained; 134 splice_donor_variants; 118 start_lost; 95 splice_acceptor_variants; 53 stop_lost; and 16 stop_retained_variants) (Table 1). The annotation also generated the functional predictions of 64,898 SNPs based on the sorting intolerant from tolerant (SIFT) algorithm [27]. There were 19,839 SNPs being deleterious and 5265 SNPs being deleterious_low_confidence. Filtering with the canonical transcripts generated 3017 deleterious SNPs (or deleterious amino acid changing SNPs; dSNPs) based on SIFT scores (<0.05).

**Figure 1:**
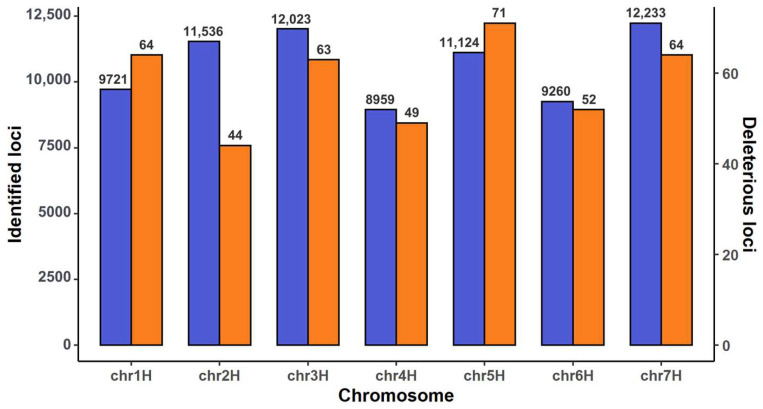
The distributions of annotated SNPs (in the blue bar) and deleterious SNPs (in the orange bar) across the seven barley chromosomes. The SNP counts are shown above the bars. Note that each barplot has its own y-axis.

**Figure 2:**
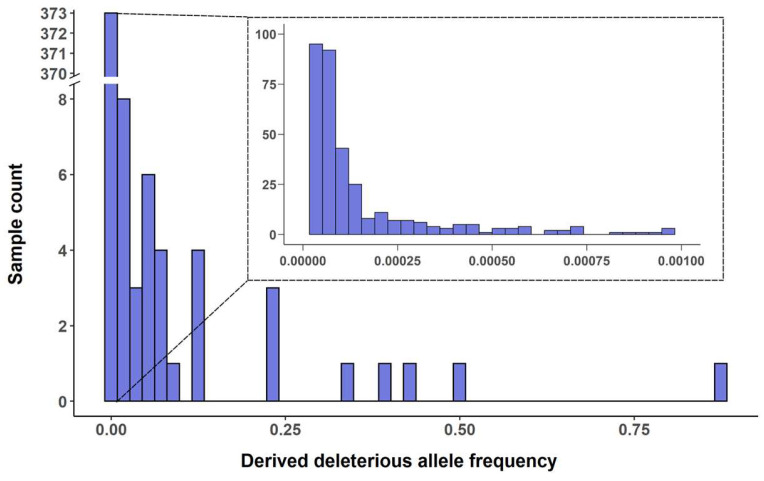
Frequency distribution of 407 deleterious SNPs in 19,778 barley samples. Note that the subfigure had 231 deleterious SNPs with allelic frequencies ranging from 0.000051 to 0.000129.

Combining SIFT scores (<0.05) with GERP++ [28] Rejected Substitution (RS) scores (RS > 0) identified 407 dSNPs, as shown in detail in Table S1 along with their gene annotations and allelic frequencies. These dSNPs were located across the seven chromosomes and each chromosome harbored 44 to 71 dSNPs (Figure 1). The dSNPs displayed an extremely skewed allelic frequency distribution in 19,778 barley samples (Figure 2). There were 22 SNPs showing occurrence frequencies of 0.05 or greater and 337 SNPs with occurrence frequencies of less than 0.001. More specifically, there were 337 dSNPs harbored in 20 or fewer barley samples (or with allelic frequencies of 0.001 or lower); 275 dSNPs occurred in five or fewer barley samples (or with allelic frequencies of 0.000253 or lower); and roughly 187 dSNPs were present only in one barley sample (or with allelic frequencies of 0.000051). Thus, a large proportion of the identified dSNPs were rare in the assayed barley samples.

### 2.2. Estimating Mutation Burdens

**Figure 3:**
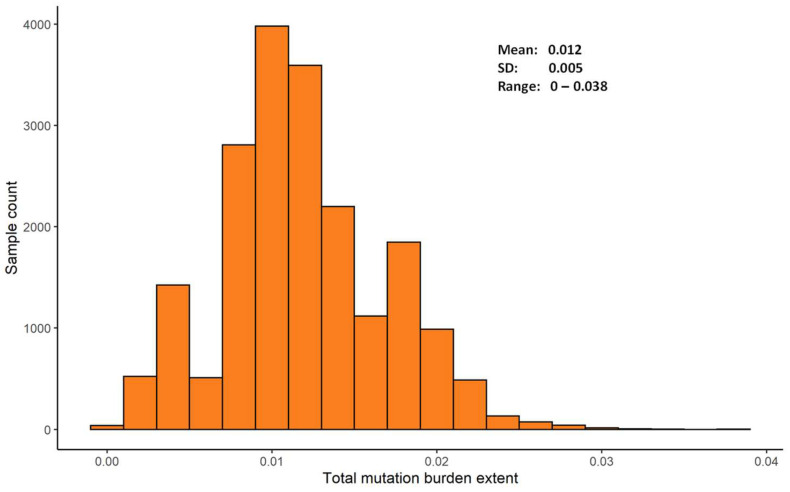
The frequency distribution of total mutation burden estimates of 19,778 barley samples.

Based on the published SNP genotype data at the 407 dSNP loci, the mutation burden per deleterious locus was estimated for individually assayed barley samples (Table S2). As expected, each sample has a mean of 0.05 deleterious heterozygotes (ranging from 0 to 14) and a mean of 4.7 deleterious homozygotes (ranging from 0 to 13). Thus, the total mutation burden estimate largely reflected the extent of the homozygous mutation burden present in the sample. The total mutation burden estimates ranged from 0 to 0.038 with a mean of 0.012 (Figure 3; Table S2). Interestingly, there were 38 barley samples with zero mutation burden estimates at the 407 predicted dSNP loci. Note that a zero mutation burden estimate in this study does not mean a sample had no, but a relatively lower count of, deleterious mutations, due to the limitation of genome sampling by the GBS method.

### 2.3. Characterizing Mutation Burdens

Characterizing the total mutation burdens estimates of the 19,778 samples revealed several patterns of total mutation burdens present in the assayed barley samples (Table 2). The analysis of variance revealed no statistically significant differences in mutation burden among the four germplasm panels, although the Chinese_Genebank panel seemed to have a higher mean burden estimate than the other panels. The mutation burden estimates differed significantly among landrace, cultivar, and breeding_material. Landrace had the highest mean burden estimate, followed by cultivar and breeding_material. Winter barley germplasm had a significantly higher mean burden estimate (0.015) than the spring barley samples (0.011). Similarly, the six-rowed barley germplasm had a significantly higher mean burden estimate (0.013) than the two-rowed barley samples (0.009), although the intermedium germplasm had the highest mean burden estimate of 0.016). Interestingly, the 1000-accession core set developed by Milner et al. [16] had a significantly higher mean burden estimate of 0.0126 than the non-core germplasm (0.0119). 

**Figure 4:**
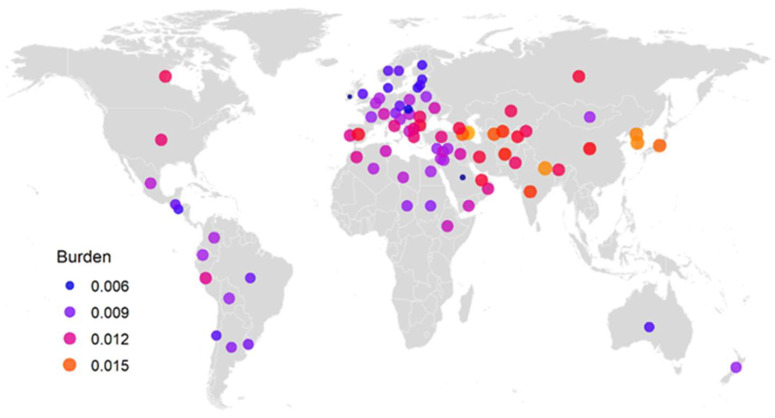
Illustration of the geographical patterns of mean mutation burden estimates of germplasm representing 84 countries. The predicted mutation burdens increased gradually toward East Asia from the center of domesticated barley origin (or Near Eastern Centre) to the East Asiatic Centre and decreased toward the North (or the European-Siberian Centre) and the South (or the Ethiopian Centre).

Further analysis of the total mutation burden estimates of the 19,778 samples for germplasm origin (or region and country) revealed significant differences in burden estimates among seven major regions (*p* < 0.0001), but not among 89 countries (*p* = 0.665). The mean burden estimates for seven regions ranged from 0.0105 to 0.0152 with an average of 0.0125 (Table 2). These mutation burdens increased gradually toward the Far East from the center of domesticated barley origin (or Near Eastern Centre; 0.0122) to the Middle Asian Centre (0.0138) and then to the East Asiatic Centre (0.0152), and they decreased toward the North (or the European-Siberian Centre; 0.0105) and toward the South (or the Ethiopian Centre; 0.0113). These three geographical patterns are illustrated in Figure 4 with the mean burden estimates representing 84 countries (with more than one sample). The mean burden estimates for 88 countries and the unknown group ranged from 0.0035 to 0.0221 with an average of 0.0113 (Table 3). Excluding the unknown groups and four countries with only one accession, the mean burden estimates for the samples of the 84 countries ranged from 0.0035 to 0.0175 with an average of 0.0112. The seven countries with the highest mean burden estimates were: Azerbaijan (0.0175), Nepal (0.0165), South Korea (0.0164), North Korea (0.0162), Japan (0.0153), Armenia (0.0152), and Turkmenistan (0.0150). The eight countries with the lowest mean burden estimates were: Ireland (0.0035), Saudi Arabia (0.0037), Slovakia (0.0057), El Salvador (0.0074), Lithuania, Estonia, Denmark, and Finland (all with 0.0075).

### 2.4. Correlating between Mutation Burdens and Averaged Pairwise Differences of Individual Samples

**Figure 5:**
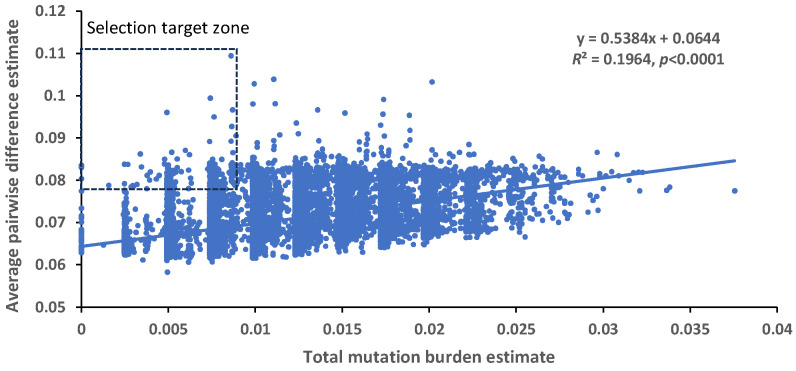
The significant association between the total mutation burden estimates generated in this study and the average pairwise difference estimates acquired from the previous study [29]. The selection target zone (in the rectangle) for germplasm with higher average pairwise difference estimates and lower total mutation burden estimates is also illustrated.

A correlation analysis between sample-wise total mutation burdens and sample-wise averaged pairwise differences (APDs) that were estimated from the same published SNP data [29] revealed a significantly positive correlation between the two estimations (Figure 5). This result is novel and interesting, as a sample with a higher mutation burden estimate had a higher average pairwise genetic difference estimate. However, for a given level of burden estimate, the correlated APD values still varied substantially.

## 3. Discussion

Our characterization took advantage of the published barley genomic data to identify 407 predicted deleterious loci across the genome and generate a novel set of mutation burden estimates for a large set of domesticated barley germplasm conserved ex situ. Analysis of the predicted mutation burdens revealed interesting patterns of mutation burden present in the conserved barley germplasm. Significant differences in burden estimate were found for several groups of barley germplasm (landrace > cultivar (or higher burden estimate in landrace than in cultivar); winter barley > spring barley; six-rowed barley > two-rowed barley; and 1000-accession core collection > non-core germplasm). Significant differences in burden estimate were also observed among seven major geographical regions. These results are useful for barley germplasm management and utilization specifically and are significant for a better understanding of the genetic risk in conserved plant germplasm in general. 

This GBS-based SNP annotation yielded 407 predicted deleterious mutations in the barley genome. The mutation count was smaller than the 1146 mutations predicted from 70 samples of a barley collection via RNA-Seq technology [8], the averaged 1000 deleterious variants per barley accession identified from exome capture sequencing data [21], and the 3855 deleterious SNPs identified from 21 barley lines by Kono et al. [30], but it was larger than the 313 mutations identified from the within-accession variation of nine barley accessions [26]. There were only 17 mutations shared between the GBS data and the RNA-Seq collection data and three mutations shared between the GBS data and the RNA-Seq accession data, while there were 223 mutations shared between the RNA-Seq collection and accession data. These differences may have reflected the effectiveness of genomic sampling via various sequencing tools and the effects of variable sample sizes and different prediction methods. For example, GBS is a method of genome complexity reduction and is expected to sample fewer deleterious loci than RNA-Seq and whole genome sequencing techniques. Using GERP++ RS (>0) scores to identify dSNPs revealed much fewer dSNPs than those from the other prediction methods (e.g., [30]), as the former considered only those SNPs in extremely constrained genic regions of the genome. However, the occurrence frequencies of the deleterious alleles identified in this study were more informative, as the sample size was large. More than 82% of the identified deleterious alleles had occurrence frequencies of 0.001 or lower (or in 20 or fewer of the 19,778 samples), and more than 67% of the deleterious alleles occurred in frequencies of 0.00025 or lower (or in five or fewer barley samples). These results clearly demonstrated the rarity of accumulative mutations in the conserved germplasm. It remains unknown, though, whether this mutational rarity was generated from germplasm conservation practices such as cold storage in the genebanks [8] or associated with the accumulative gene loss from barley domestication [17] and/or barley genome evolution similar to other grass genomes (e.g., see [31,32,33]). Also, the prediction of deleterious SNPs could be biased from paralogous genes, if any, in the barley genome. 

Our mutation burden estimation is dependent on the genomic prediction of the deleterious mutations with amino acid substitutions without empirical validations of their actual impacts on plant fitness and the frequency of the predicted deleterious alleles. Thus, caution is required in the interpretation of the predicted deleterious mutations and estimated mutation burdens. Genomic mutation prediction and mutation burden estimation can vary among the studies of variable sample sizes with different genomic sampling. For example, the GBS-based estimates of the total mutation burden ranged from 0 to 0.038 with a mean of 0.012 (Figure 3). The same estimates from the RNA-Seq collection data of 70 barley samples ranged from 0.146 to 0.198 with a mean of 0.176 [8] and those from RNA-Seq data of nine accessions ranged from 0.541 to 0.747 with a mean of 0.681 [26]. Research is needed to analyze the effects of various factors such as genomic data type and sample size on mutation burden estimation. Also, it is worth noting that our estimated mutation burdens can be biased by inaccurate SNP genotyping [24] and the imputation of missing SNP genotypes for the average missing call of 2.9% per marker [16]. It is difficult to assess the extent of such prior biases in our burden estimation. Moreover, the sample-wise mutation burden estimate may not fully represent its representative barley accession, as within-accession genetic variation exists (e.g., [16,26]). These limitations taken together call for further research to improve the estimation of mutation burdens in conserved germplasm.

The new finding of significant differences in the estimated burden of several paired groups of barley germplasm (landrace > cultivar; winter barley > spring barley; six-rowed barley >two-rowed barley; and 1000-accession core collection > non-core germplasm) is interesting, but not surprising. These differences may have reflected the differences in genetic diversity between two paired germplasm groups, as there was a strong, positive correlation between sample-wise mutation burdens and the estimates of sample APDs for genetic distinctness (Figure 5). The higher genetic distinctness a barley sample has, the higher the mutation burden. This is understandable, as a group of the genetically diverse germplasm like the core collection has a high chance of carrying more unique alleles (or higher genetic diversity), some of which may be deleterious in nature (or have a higher predicted mutation burden). The germplasm groups of core collection, landrace, six-rowed, and winter barley are all expected from previous diversity studies [16,34] to have more genetic diversity and they also displayed higher burden estimates than their paired groups in this study (Table 2). Also, the finding that the landrace group had a higher burden estimate in landrace than the cultivar group was not consistent with the report by Fu [26], but matched well with the previous reasoning from other studies [21,24,35] that a cultivar generally has more direct selection on yield and can purge more genetic load than a landrace. 

Current genomic characterization also revealed an interesting, novel finding that the predicted mutation burdens were strongly associated with the geographic origins of barley germplasm (Table 2). The predicted mutation burdens increased gradually toward East Asia from the center of domesticated barley origin (or Near Eastern Centre) to the East Asiatic Centre and decreased toward the North (or the European-Siberian Centre) and the South (or the Ethiopian Centre) (Figure 4). It is difficult to explain biologically such geographic patterns of mutation burden, as there were no statistically significant differences in mutation burden at the country level. However, it may imply that the mechanisms for mutation accumulation and purging might differ in barley grown in different geographical regions. Comparative research may be needed to investigate barley mutation accumulation and purging in different geographical regions using a whole genome sequencing technique.

The revealed patterns of the predicted mutation burdens are useful for barley germplasm management and utilization. For example, more management effort such as viability monitoring may be needed on the germplasm of landrace, winter barley, six-rowed barley, and core set than those accessions with the lower predicted mutation burdens. Similarly, frequent germination testing may be conducted on those barley accessions originating from those countries with the highest mean burden estimates such as Azerbaijan, Nepal, South Korea, North Korea, and Japan. However, the positive correlation between sample-wise mutation burdens and the estimates of sample APDs for genetic distinctness (Figure 5) could impose some difficulty in germplasm choice for use [36], as barley breeders would target the germplasm with more genetic distinctness and lower (but not higher) mutation burden. Thus, caution is needed in germplasm selection for its use by considering both genetic diversity and mutation burden. One workable option for barley breeding is to select germplasm with the genetic distinctness as high as possible and the mutation burden as low as possible, as illustrated in Figure 5, with a target selection zone of germplasm with higher genetic distinctness and lower mutation burden estimates for the assayed 19,778 samples. This option would increase the chance of selecting many of the 38 barley samples with zero mutation burden estimates at the 407 deleterious SNP loci. More interestingly, these 38 samples were collected from 14 countries and represented two-rowed and six-rowed kernel types of landrace, cultivars, and breeding materials with spring and winter growth habits (Table S2). 

Our findings, along with those published at the collection and accession levels [8,26], form an important part of baseline information on deleterious mutations present within and among conserved germplasm collections. This information is essential for understanding the genetic cost of long-term germplasm conservation and for our search for mitigating strategies to minimize the impact of deleterious mutations on conserved germplasm, as this is a risk of declining fitness in conserved germplasm. Now it has become much more clear that genomic tools can facilitate the mutation screening of the conserved germplasm; the extent of deleterious mutations varies within and among germplasm collections; and a majority of deleterious alleles exist at a low frequency. Thus, genetic risk exists in the conserved barley germplasm and the need is warranted to develop effective conservation strategies for minimizing the within-genebank genetic erosion and vulnerability from deleterious mutations at the collection and accession levels. New management procedures such as the mutation screening of conserved germplasm are required, and/or related management procedures such as viability testing [37] may need to be modified for genebank operations [38,39]. However, several lines of research will be helpful for the development of better long-term conservation strategies. First, empirical research is needed on the fitness consequence of the predicted deleterious mutations on the conserved germplasm [40], as the reported mutation burdens were predictive in nature, although evidence exists that phenotypic mutations were induced during storage in barley and pea seeds [7]. Second, more research on the association of the predicted mutation burdens with the long-term conservation practices in genebanks such as storage condition and year, viability tests, and germplasm regeneration can inform the development of effective mitigating strategies. Third, our GBS-based burden estimates are encouraging, as the low genome-wide coverage of GBS is still informative to extrapolate mutational differences between samples to the genome. However, the optimization of deleterious mutation assessments with more informative genomic tools such as whole genome sequencing and effective germplasm sampling can allow for more efficient mutation screening of conserved germplasm to yield a more informative estimation of mutation burdens and to analyze the genetic risks present in many other germplasm collections.

## 4. Materials and Methods

Materials analyzed for this study were 19,778 domesticated barley accessions, which were part of 22,626 barley samples previously characterized by Milner et al. [16] via GBS. The domesticated barley germplasm consisted of four germplasm panels: 18,714 accessions conserved in IPK Gatersleben; 669 accessions of the Swiss National Genebank of Agroscope; 257 accessions of the barley collection of the National Crop Genebank of China at the Institute of Crop Sciences of the Chinese Academy of Agricultural Sciences; and 138 diverse, largely georeferenced accessions assayed previously by Pourkheirandish et al. [41] (Table S2). Milner et al. [16] called SNPs from cleaned GBS sequence reads based on the reference genome sequence of barley cultivar Morex [42] and generated a panel of 76,102 SNP genotypes for 19,778 domesticated barley samples each representing a barley accession (180606_GBS_domesticated_barley_19778_samples_76102_SNPs.vcf.gz). This SNP data set, along with the passport data set, was acquired following the instructions described in the sections of Data Availability and Supplementary Information from the journal website. These data sets formed the basis for predicting the mutation burden for each barley sample in this study.

The SNP data set was annotated using the stand-alone Ensembl Variant Effect Predictor (VEP) with the cache data of hordeum_vulgare_vep_40_Hv_IBSC_PGSB_v2.tar.gz [43,44]. The deleterious effect of a nonsynonymous SNP variant was predicted using the SIFT algorithm [27] and each SNP was annotated with a SIFT score. The SIFT score predicts the impact of an amino acid substitution and can distinguish between functionally neutral and deleterious amino acid changes. An amino acid substitution with a SIFT score of 0.05 or less is considered to be deleterious. In our companion research using the same version of the reference genome sequence of barley cultivar Morex [8], the phylogenetic position-specific constraint from the substitution of a locus [12] was inferred using GERP++ [28] and the GERP++ RS scores were generated for the barley reference genome based on 12 reference genomes of other plant species (i.e., wheat, brome, millet, rice, maize, sunflower, grape, soybean, bean, thale, rapa, and banana) [8]. Specifically, these scores were obtained by comparing the level of substitution observed to that expected if there was no functional constraint to identify constrained loci in multiple sequence alignments. A positive score represents a highly-conserved position while a negative score represents a highly-variable position. Thus, a substitution occurring at a site with RS > 0 (or at a highly-conserved position) is predicted to be deleterious; the larger the RS score, the more deleterious the substitution. In this study, the GERP++ RS (>0) scores (or barley-RS-scores.gtzero.txt.gz) generated by Fu et al. [8] were combined with SIFT (<0.05) scores to identify dSNPs in the constrained portions of the genome. 

With the predicted dSNPs, mutation burden per deleterious locus for an individual sample was estimated from the sample SNP genotype data based on the numbers of deleterious alleles present in three models: homozygous mutation burden, heterozygous mutation burden, and total mutation burden [15,45]. The homozygous mutation burden per deleterious locus is the number of derived deleterious alleles in the homozygous state, divided by a product of 2× the total dSNP count. The heterozygous mutation burden per deleterious locus is the number of derived deleterious alleles existing in the heterozygous state, divided by a product of 2× the total dSNP count. The total mutation burden per deleterious locus is the number of derived deleterious alleles existing in an accession (2× homozygous mutation burden + heterozygous mutation burden), divided by a product of 2× the total dSNP count. 

To better characterize the predicted mutation burdens estimated for 19,778 samples, further analyses were made in several aspects to assess the patterns of the predicted mutation burdens. First, the distributions of the identified dSNPs across the seven chromosomes and the occurrence frequencies of the predicted deleterious alleles in all the assayed samples were assessed. Second, the distribution of the predicted total mutation burdens in all the barley samples was analyzed. Third, the basic statistics of the sample-wise total mutation burdens were calculated for different barley groups: (1) four germplasm panels; (2) three germplasm types; (3) three growth habits; (4) different kernel types; (5) country origins; (6) geographical regions; and (7) core vs non-core sets. Analysis of variance was applied to test the significance of the differences in mutation burden for each barley group. The grouping of barley germplasm was based on the inventory information present in the passport data of 19,778 samples [16]. Extra germplasm grouping into seven geographical regions was made following the agri-ecological classification of cultivated barley described by Knüpffer et al. [46]. Fourth, a correlation was also made between the estimates of total mutation burdens and APDs of individual samples. The APD data (or Hordeum-vulgare-passport-APD.xlsx) was previously generated from the analysis of genetic distinctness and redundancy for 19,778 barley samples based on the same SNP genotype data set [29]. The larger the APD value, the more genetically distinct the sample is from the other samples, allowing for the measurement of sample genetic distinctness or redundancy [47]. 

## 5. Conclusions

Predicting sample-wise mutation burdens for 19,778 domesticated barley germplasm revealed 407 deleterious mutations harbored in the assayed samples and 337 (or 82%) identified deleterious alleles present in 20 (or 0.1%) or fewer barley accessions. Significant differences in burden estimate were found for several groups of barley germplasm (landrace > cultivar (or higher burden estimate in landrace than in cultivar); winter barley > spring barley; six-rowed barley >two-rowed barley; and 1000-accession core collection > non-core germplasm). Significant differences in burden estimate were also observed among seven major geographical regions. The sample-wise predicted mutation burdens were positively correlated with the estimates of sample-wise average pairwise genetic difference. These findings are significant for barley germplasm management and utilization and for a better understanding of the genetic risk in conserved plant germplasm. 

## Figures and Tables

**Table 1:** Annotation results of 76,102 barley SNPs generated by Milner et al. [16].

SNP Annotation	Count
*VEP consequence type*	*74,856*
missense_variant	12,783
splice_region_variant	854
stop_gained	356
splice_donor_variant	134
start_lost	118
splice_acceptor_variant	95
stop_lost	53
stop_retained_variant	16
synonymous_variant	8544
5_prime_UTR_variant	4752
3_prime_UTR_variant	4644
non_coding_transcript_exon_variant	1350
intron_variant	9726
upstream_gene_variant	6585
downstream_gene_variant	5645
intergenic_variant	19,201
	
*SIFT prediction*	*64,898*
deleterious	19,839
deleterious_low_confidence	5265
tolerated	33,699
tolerated_low_confidence	6095

**Table 2:** Statistics and ANOVA tests for total mutation burden estimates of 19,778 barley samples for six barley germplasm groups.

Group	Count	Mean	SD	Min	Max	ANOVA
*Germplasm_panel*						*p* = 0.9504
IPK_Genebank	18,714	0.0119	0.0049	0.0000	0.0376	
Chinese_Genebank	257	0.0158	0.0071	0.0000	0.0323	
Swiss_Genebank	669	0.0113	0.0038	0.0025	0.0205	
Pourkheirandish_et_al_2015	138	0.0113	0.0042	0.0025	0.0223	
*Material_type*						*p* < 0.00001
Landrace	12,792	0.0122	0.0044	0.0000	0.0323	
Cultivar	5118	0.0115	0.0056	0.0000	0.0376	
Breeding_material	1864	0.0112	0.0054	0.0000	0.0273	
Unknown	4	0.0105	0.0012	0.0099	0.0123	
*Growth_habit*						*p* < 0.00001
Spring	14,643	0.0110	0.0046	0.0000	0.0323	
Intermediate	152	0.0146	0.0045	0.0049	0.0253	
Winter	3920	0.0151	0.0048	0.0000	0.0376	
Unknown	1063	0.0115	0.0040	0.0025	0.0272	
*Row_type*						*p* < 0.00001
Two-rowed	3819	0.0092	0.0044	0.0000	0.0250	
Intermedium	347	0.0162	0.0049	0.0024	0.0272	
Six-rowed	7821	0.0134	0.0046	0.0000	0.0376	
Deficiens	696	0.0117	0.0032	0.0025	0.0209	
Labile	242	0.0120	0.0022	0.0074	0.0201	
Unknown	6853	0.0115	0.0049	0.0000	0.0278	
*1000-accession_core_set*						*p* < 0.00001
Core	998	0.0126	0.0051	0.0000	0.0280	
Non-core	18,780	0.0119	0.0049	0.0000	0.0376	
*Region*						*p* < 0.00001
Near Eastern Centre	1850	0.0122	0.0042	0.0024	0.0294	
Mediterranean Centre	2327	0.0129	0.0046	0.0000	0.0321	
Middle Asian Centre	857	0.0138	0.0049	0.0025	0.0296	
East Asiatic Centre	2490	0.0152	0.0052	0.0000	0.0376	
European-Siberian Centre	5021	0.0105	0.0050	0.0000	0.0297	
Ethiopian Centre	3952	0.0113	0.0031	0.0000	0.0271	
New World Centre	1024	0.0120	0.0052	0.0000	0.0271	
Unknown	2257	0.0105	0.0052	0.0000	0.0271	

**Table 3:** Statistics of the total mutation burden estimates for the barley samples of 88 countries (and the unknown group) representing seven regions. NE = Near Eastern Centre; M = Mediterranean Centre; MA = Middle Asian Centre; EA = East Asiatic Centre; ES = European-Siberian Centre; E = Ethiopian Centre; NW = New World Centre; and NA is not available for four countries with only one accession.

Country	Region	Count	Mean	SD	Min	Max
Ireland	ES	5	0.00346	0.00136	0.00246	0.00498
Saudi Arabia	M	2	0.00369	0.00173	0.00246	0.00491
Slovakia	ES	162	0.00574	0.00224	0.00000	0.01605
El Salvador	NW	2	0.00744	0.00341	0.00503	0.00985
Lithuania	ES	3	0.00747	0.00260	0.00493	0.01013
Estonia	ES	4	0.00748	0.00205	0.00498	0.01000
Denmark	ES	123	0.00753	0.00369	0.00246	0.02010
Finland	ES	59	0.00753	0.00229	0.00491	0.01478
Costa Rica	NW	1	0.00756	NA	0.00756	0.00756
Australia	NW	48	0.00777	0.00324	0.00246	0.01489
Latvia	ES	5	0.00790	0.00269	0.00493	0.01232
UK	ES	211	0.00796	0.00516	0.00000	0.02970
Chile	NW	12	0.00798	0.00458	0.00491	0.01609
Norway	ES	10	0.00813	0.00234	0.00491	0.01238
Sweden	ES	196	0.00819	0.00407	0.00000	0.02100
Guatemala	NW	8	0.00832	0.00127	0.00737	0.00990
Czechia	ES	29	0.00835	0.00397	0.00246	0.02020
Brazil	NW	2	0.00867	0.00184	0.00737	0.00998
Uruguay	NW	27	0.00904	0.00353	0.00246	0.01737
Austria	ES	252	0.00949	0.00423	0.00246	0.02222
Argentina	NW	25	0.00959	0.00432	0.00248	0.02057
Sudan (the)	E	11	0.00971	0.00224	0.00503	0.01250
South Africa	E	1	0.00993	NA	0.00993	0.00993
Chad	E	20	0.00993	0.00295	0.00250	0.01746
Cyprus	M	6	0.01005	0.00489	0.00495	0.01728
New Zealand	NW	2	0.01010	0.01428	0.00000	0.02020
Germany	ES	1415	0.01026	0.00560	0.00000	0.02519
Belarus	ES	7	0.01029	0.00327	0.00495	0.01478
France	ES	267	0.01038	0.00507	0.00000	0.02793
Mongolia	EA	33	0.01045	0.00334	0.00491	0.01733
Bolivia	NW	35	0.01048	0.00424	0.00246	0.01852
Ecuador	NW	14	0.01057	0.00355	0.00246	0.01724
Egypt	M	32	0.01062	0.00364	0.00491	0.01970
Syria	NE	149	0.01066	0.00365	0.00248	0.02020
Jordan	NE	26	0.01068	0.00345	0.00318	0.01605
Colombia	NW	22	0.01071	0.00376	0.00493	0.01985
Netherlands	ES	88	0.01071	0.00574	0.00246	0.02256
Croatia	ES	7	0.01073	0.00383	0.00491	0.01519
Algeria	M	49	0.01081	0.00359	0.00246	0.01980
Belgium	ES	27	0.01082	0.00606	0.00249	0.02211
Israel	NE	51	0.01086	0.00515	0.00246	0.02239
Poland	ES	166	0.01090	0.00573	0.00246	0.02356
Hungary	ES	84	0.01094	0.00471	0.00254	0.02228
Mexico	NW	26	0.01096	0.00363	0.00254	0.01500
Libya	M	162	0.01098	0.00348	0.00248	0.02036
Albania	ES	52	0.01124	0.00390	0.00249	0.01852
Lebanon	NE	10	0.01128	0.00123	0.00983	0.01244
Ethiopia	E	3919	0.01135	0.00312	0.00000	0.02709
Tunisia	M	56	0.01136	0.00385	0.00493	0.02015
Yemen	M	56	0.01140	0.00210	0.00493	0.01720
Ukraine	ES	100	0.01151	0.00342	0.00493	0.01980
Switzerland	ES	786	0.01151	0.00395	0.00246	0.02513
Iraq	NE	71	0.01171	0.00439	0.00249	0.02681
Morocco	M	111	0.01202	0.00348	0.00248	0.02073
Portugal	M	17	0.01206	0.00517	0.00491	0.01857
Peru	NW	29	0.01210	0.00316	0.00739	0.01741
Oman	M	10	0.01210	0.00337	0.00494	0.01481
Italy	M	324	0.01223	0.00467	0.00000	0.02730
Turkey	NE	1383	0.01234	0.00414	0.00240	0.02941
Eritrea	E	1	0.01235	NA	0.01235	0.01235
Macedonia	M	2	0.01236	0.00002	0.01235	0.01238
Greece	M	328	0.01255	0.00419	0.00262	0.02463
USA	NW	605	0.01259	0.00509	0.00000	0.02709
Romania	ES	105	0.01276	0.00534	0.00247	0.02519
Pakistan	M	321	0.01282	0.00466	0.00372	0.02757
Kyrgyzstan	MA	4	0.01296	0.00548	0.00491	0.01720
Kazakhstan	MA	7	0.01303	0.00341	0.00739	0.01728
Canada	NW	166	0.01303	0.00616	0.00246	0.02604
Bhutan	EA	10	0.01312	0.00160	0.01013	0.01538
Georgia	NE	125	0.01327	0.00379	0.00256	0.02217
Iran	MA	422	0.01345	0.00487	0.00246	0.02672
Russia	ES	217	0.01354	0.00399	0.00246	0.02481
United Arab Emirates	M	2	0.01357	0.00519	0.00990	0.01724
Bulgaria	ES	143	0.01358	0.00396	0.00493	0.02239
Tajikistan	MA	10	0.01361	0.00456	0.00985	0.02233
Spain	M	170	0.01373	0.00418	0.00491	0.02302
China	EA	806	0.01396	0.00584	0.00000	0.03226
Afghanistan	MA	345	0.01410	0.00501	0.00248	0.02778
Uzbekistan	MA	38	0.01424	0.00414	0.00493	0.02463
India	M	679	0.01433	0.00510	0.00000	0.03210
Turkmenistan	MA	31	0.01501	0.00602	0.00249	0.02965
Armenia	NE	22	0.01516	0.00423	0.00761	0.02006
Japan	EA	722	0.01531	0.00502	0.00000	0.03756
North Korea	EA	116	0.01619	0.00405	0.00000	0.02799
South Korea	EA	249	0.01637	0.00353	0.00513	0.02744
Nepal	EA	554	0.01652	0.00472	0.00293	0.02750
Azerbaijan	NE	13	0.01754	0.00510	0.00739	0.02228
Moldova	ES	1	0.02211	NA	0.02211	0.02211
Unknown		1888	0.01064	0.00522	0.00000	0.02709

## Data Availability

Some supplemental output or metadata can be found in the Supplementary Materials.

## Supplementary Materials

The following supporting material can be found online at Figshare DOI (https://doi.org/10.6084/m9.figshare.25241464 (accessed on 24 May 2024)): Table S1 List of 407 deleterious SNPs, VEP-annotations and allelic frequencies. Table S2 List of total mutation burden estimates for 19,778 IPK domesticated barley samples.
